# The prevalence, risk factors and lifestyle patterns of Jordanian females with premenstrual syndrome: a cross-sectional study

**DOI:** 10.2144/fsoa-2023-0056

**Published:** 2023-08-08

**Authors:** Mera A Ababneh, Malak Alkhalil, Abeer Rababa'h

**Affiliations:** 1 Department of Clinical Pharmacy, Faculty of Pharmacy, Jordan University of Science & Technology, Irbid, 22110, Jordan

**Keywords:** A-PMS scale, behavior, diet, emotional, herbal, lifestyle, physical, premenstrual syndrome, psychological, risk factors

## Abstract

**Aim:**

The study aims to assess factors associated with premenstrual syndrome (PMS) and the frequency of using painkillers to relieve premenstrual pain.

**Methods:**

This is a cross-sectional study of 1580 premenopausal women. An online self-administered questionnaire consists of sociodemographics, and the diagnostic criteria using the Arabic Premenstrual Syndrome Scale (A-PMS).

**Results:**

The prevalence of PMS among Jordanian females was 94%. Moreover, a significant correlation was found between several factors, including BMI, family history of PMS, smoking, and herbal tea consumption and the psychological, physical and behavioral symptoms of PMS. Furthermore, analgesic use for pain relief and food cravings were significantly associated with psychological, physical and behavioral PMS symptoms.

**Conclusion:**

PMS is highly prevalent and affects women in different life aspects.

## Plain language summary

Premenstrual syndrome (PMS) is a set of symptoms that affect women for a week or two before their period. PMS influences women's emotions, physical health and behavior. Symptoms usually resolve within a few days of menstruation. This study confirms the epidemiological surveys worldwide that reported 80–90% of females experience PMS symptoms. We reported multiple risk factors for PMS including age, weight, family history of PMS, marital status, smoking, herbal tea consumption, fast food consumption and other dietary habits. Furthermore, analgesic use for pain relief and food cravings were significantly associated with psychological, physical and behavioral PMS.

Premenstrual syndrome (PMS) is a group of symptoms that affect many women for a week or two before their menses, thus affecting their emotions, physical health and behavior during the luteal phase of the menstrual cycle and resolving within a few days [1].

PMS is diagnosed based on several criteria, including the International Classification of Disease (ICD-10), the American College of Obstetricians and Gynecologists (ACOG) and the Diagnostic and Statistical Manual of Mental Disorders (DSM-IV) [2,3]. As with other syndromes, etiology is not fully understood. However, various factors contribute to the etiology, including genetic predisposition, ethnicity and hormonal fluctuations [4].

A growing body of literature has evaluated PMS and found that women with PMS have cyclic fluctuations in ovarian steroid levels, therefore causing unpredictable changes in opioid levels, the GABAergic system modulated by progestin, and eventually the serotonin levels [5,6]. According to the most recent studies, it is found that there is a high prevalence of PMS symptoms in women. Epidemiological surveys have found that about 80–90% of females experience PMS symptoms and about 5% experience severe symptoms that interfere with their normal activities [7]. Even though clinicians do not diagnose PMS as a medical condition, rising PMS levels would increase healthcare utilization. Thus, public clinicians should learn more about this serious condition [8].

A key problem that in case people overlook PMS diagnosis, this would result in a modest increase in direct medical costs with an average annual growth of $59 in direct costs (p < 0.026) and a significant increase in indirect costs of about $4333 per patient (p < 0.0001) compared with patients without PMS [9]. Therefore, this study aimed to measure the prevalence of PMS among Jordanian women and explore lifestyle factors and dietary habits associated with PMS.

## Methodology

### Design & setting

A cross-sectional study was conducted on Jordanian women with menses aged between 18 and 50 years from 15 November 2021 to 15 February 2022. Convenience and snowball sampling were used. An online multiple-choice questionnaire was administered via Google Forms and disseminated through social media.

### Inclusion & exclusion criteria

Participants in this study included women aged between 18 and 50 years who had a menstrual period within the last 2 months. Exclusion criteria included women taking psychopharmacological medicines (e.g., antidepressants, antipsychotics), hormonal replacement therapy, lactation within 3 months before the study, pregnancy and oral contraceptive use.

### Study questionnaire

A structured questionnaire was created and tested on a pilot population to improve the design and check the questionnaire's feasibility and clarity. The participants in the pilot study were eliminated from the final data analysis.

The questionnaire's final version comprised two sections and was self-administered online. The first section consisted of sociodemographic data such as age, level of education, occupation working in the health field or not and geographic area. In addition, more details were needed, such as the age at menarche, marital status, number of pregnancies and number of children. Also, anthropometric measurements were required. Based on height and body mass weight, body mass index was calculated using the Quetelet equation (body mass (kg)/height (m^2^)) and interpreted according to the criteria designated by the WHO [10].

Furthermore, in light of the data that supports PMS's relation to genetic inheritance, an additional question about PMS’ family history was presented. Last, The first section ended with the Arabic Premenstrual Syndrome Scale (A-PMS) [11]. Regarding similar tools, Al-Gahtani and Jahrami developed the first tool based on DSM-IV-TR criteria to screen and evaluate the severity of PMS among Arabic-speaking women.

A total of 23 items were divided into three domains: physical symptoms, psychological symptoms and impairment of functioning. Each symptom on the scale was categorized as (none [0], mild [1], moderate [2] and severe [3]). The following psychological symptoms were examined (depressed mood, feeling hopelessness, feeling guilty, anxiety, mood fluctuation, increased sensitivity toward others, anger, easily tempered, decrease or lack of interest, difficulty concentrating, lethargy, insomnia, sense of loss of control and feeling overwhelmed, etc.). Additionally, many physiological symptoms like breast tenderness, headache, muscle or joint pain and acne were also evaluated [11,12].

For lifestyle evaluation such as smoking were examined (current smoker, never a smoker, secondhand smoker or former smoker, for how long she has quit), the type of smoke (cigarettes, cigars, or shisha (hookah) or pipe (vape) which could be a contributing factor.

In addition, this study examined dietary habits among participants. We asked about the consumption of carbohydrates, healthy fats, dairy products, fruits, leafy vegetables, fast food, coffee and tea and herbal products. Supplement consumption was evaluated in the last 3 months, including (vitamin D, calcium, multivitamin, omega-3 zinc-vitamin C, other supplements or none). Furthermore, participants were asked about their dietary and lifestyle habits before or during their PMS. Finally, participants were asked about the use of pain painkillers in their menstrual cycle.

### Data analysis

Descriptive analysis was illustrated as the mean and standard deviation for continuous data. In contrast, frequencies and percentages were used to summarize categorical data and present PMS symptoms' severity. The Pearson correlation coefficient was used to explain the correlation between physiological, psychological and behavioral PMS scores. In each domain, participants who provided a “none” response were deemed to have no PMS, while mild, moderate and severe responses were added and grouped as participants with PMS.

The prevalence of PMS symptoms among females scored 1485 out of 1580 responses, representing 94%. Based on the average score, we computed the score of each domain using a transformation procedure. After each domain was scored, its scores were converted into categories. The four types are: [0 to <1] refers to no PMS, [>1 to <2] refers to mild PMS, [>2 to <3] refers to moderate PMS, and [>3- to 4] refers to severe symptoms PMS. Multivariable logistic regression analysis determined independent risk factors correlated with PMS for each score category. All tests performed were two-tailed significance tests and a p-value (p = <0.05) was considered significant. Statistical analysis was performed using SPSS version 23 (IBM Corp., USA).

## Results

This study included 1580 participants with a mean age of 27.3 years ±6.7. The mean BMI was 24.3 ± 4.7 kg/m^2^. More than half of the respondents' residences were from the middle region 65.8%. Furthermore, 40% were mainly employees, and 34.6% of the participants were not in the health field. The vast majority (86.4%) of participants had menarche at age ≥12 years. Below three quarters (71.1%) of the participants were single, and (28.9%) were married. In addition, participants were asked if they have a history of PMS in their families, and results showed that two-thirds (71.5%) of the participants have never had any PMS history. Other demographics of the study sample are indicated in Table 1.

**Table 1.** Participants' sociodemographic characteristics (n = 1580).

Characteristics	n (%)
Age (year) Mean ± SD	27.3 ± 6.7
BMI Mean ± SD	24.3 ± 4.7
Occupation Employed Unemployed Retired Student	640 (40.5%) 483 (30.6%) 11 (0.7%) 446 (28.2%)
Work in the health field Yes No Unemployed	305 (19.3%) 546 (34.6%) 729 (46.1%)
Place of residence North region Middle region South region	479 (30.3%) 1039 (65.8%) 62 (3.9%)
Educational level Primary/secondary education Bachelor Diploma Postgraduate	82 (5.2%) 1075 (68.1%) 113 (7.2%) 309 (19.6%)
Marital status Single/divorced/widowed Married	1124 (71.1) 456 (28.9%)
Pregnancies Once Twice Thrice More than 3 Not married None	104 (6.6%) 98 (6.2%) 88 (5.6%) 116 (7.3%) 1075 (68.0%) 99 (6.3%)
Children One child Two children Three children More than three children None Not married	103 (6.5%) 115 (7.3%) 86 (5.4%) 79 (5.0%) 118 (7.5%) 1079 (68.3%)
Age of menarche ≥12 years <12 years	1365 (86.4%) 215 (13.6%)
Family history of premenstrual syndrome No Yes	1130 (71.5%) 450 (28.5%)

SD: Standard deviation.

Among the study participants, the prevalence of PMS was 94% and the prevalence of each premenstrual symptom (classified according to severity) is shown in Table 2. Overall, the most frequently reported premenstrual symptoms were depressed mood (45.7%), followed by muscle, joint, abdominal and back pain (43%) and anger feelings (42.3%). Nevertheless, the most frequently demonstrated severe physical symptom was muscle, joint, abdominal and back pain (43%). In comparison, anger feelings (42.3%), affective labiality (40.4%) and increased sensitivity toward others (40.1%) were the most often reported severe psychological symptoms.

**Table 2.** Prevalence of premenstrual syndrome symptoms by the level of severity (n = 1580).

Lifestyle characteristics	n (%)
Smoking Current smoker Never smoker Former smoker Secondhand smoker	367 (23.2%) 893 (56.5%) 28 (1.8%) 292 (18.5%)
Type of smoking Cigarettes Cigar Waterpipe Vape Not a smoker	110 (7.0%) 3 (0.2%) 293 (18.5%) 25 (1.6%) 1149 (72.7%)
Average of cigarettes/cigars per day ≥15 cigarettes/cigar per day less than 15 cigarettes/cigars per day Not a smoker	51 (3.2%) 126 (8.0%) 1403 (88.8%)
Water pipe/vape per day Daily 2–3 per week Once per week 2–3 per month I am not a smoker	136 (8.6%) 86 (5.4%) 66 (4.2%) 104 (6.6%) 1188 (75.2%)
Quit smoking since Ex-smoker <12 months Ex-smoker >12 months I am not a smoker I am a current smoker	29 (1.8%) 16 (1.0%) 1230 (77.9%) 305 (19.3%)
Physical exercise (30 min) No exercise 1–2/week 3–5-times/week Daily	849 (53.7%) 430 (27.2%) 223 (14.2%) 78 (4.9%)
Type of exercise Aerobic/cardio exercise Weightlifting Yoga None	660 (41.8%) 48 (3.1%) 37 (2.3%) 835 (52.8%)
Sleep duration Less than 6 h/day 6–8 h/day More than 8 h/day	241 (15.3%) 1100 (69.6%) 239 (15.1%)

SD: Standard deviation.

As demonstrated in Table 2, commonly reported moderate symptoms were depressed mood (45.7%), noting that depression was not clinically diagnosed scale but self-reported; anxiety/worry (37.8%), and feeling overwhelmed (37.5%). While headache and acne (30.3% and 30.2%) are the most documented mild symptoms. Interestingly, most participants reported that “moderate behavioral symptoms” was found to interfere with relationships with (31.0%), work or school (44.1%) and daily routines (43.6%). Other responses are listed in Table 2.

Table 3 reveals responses to lifestyle questions. As for the smoking status, more than half of the participants were non-smokers, and slightly less than a quarter were smokers. However, when the participants were asked how often they exercise, almost half responded negatively, ‘I don't do exercise’ (53.7%). In addition, participants were asked about sleeping duration; more than two-thirds (69.6%) of the participants slept 6–8 h per day.

**Table 3.** Participants' lifestyle characteristics and dietary habits (n = 1580).

Lifestyle characteristics	n (%)
Smoking Current smoker Never smoker Former smoker Secondhand smoker	367 (23.2%) 893 (56.5%) 28 (1.8%) 292 (18.5%)
Type of smoking Cigarettes Cigar Waterpipe Vape Not a smoker	110 (7.0%) 3 (0.2%) 293 (18.5%) 25 (1.6%) 1149 (72.7%)
Average of cigarettes/cigars per day ≥15 cigarettes/cigar per day less than 15 cigarettes/cigars per day Not a smoker	51 (3.2%) 126 (8.0%) 1403 (88.8%)
Water pipe/vape per day Daily 2–3 per week Once per week 2–3 per month I am not a smoker	136 (8.6%) 86 (5.4%) 66 (4.2%) 104 (6.6%) 1188 (75.2%)
Quit smoking since Ex-smoker <12 months Ex-smoker >12 months I am not a smoker I am a current smoker	29 (1.8%) 16 (1.0%) 1230 (77.9%) 305 (19.3%)
Physical exercise (30 min) No exercise 1–2/week 3–5-times/week Daily	849 (53.7%) 430 (27.2%) 223 (14.2%) 78 (4.9%)
Type of exercise Aerobic/cardio exercise Weightlifting Yoga None	660 (41.8%) 48 (3.1%) 37 (2.3%) 835 (52.8%)
Sleep duration Less than 6 h/day 6–8 h/day More than 8 h/day	241 (15.3%) 1100 (69.6%) 239 (15.1%)

Regarding dietary habits, most participants reported consuming starchy foods and dairy products daily (44.6 and 44.0%, respectively). In addition, responses were almost similar about how many servings of fruits and leafy vegetables they consume/day; most of them answered 1–2 servings/day (46.9 and 45.5%, respectively). Interestingly, the consumption of caffeine-containing beverages (coffee and tea) was measured to be 1–2 cups a day (51.3%, 34.1%). Other responses of participants were summarized in Table 4.

**Table 4.** Participants' daily dietary behaviors and dietary behaviors before/during premenstrual syndrome.

Daily dietary behaviors	n (%)
Complex carbohydrates 1–2 per month 1–2 per week 3–5 per week Daily I don't eat this type of food	206 (13.0%) 311 (19.7%) 310 (19.6%) 704 (44.6%) 49 (3.1%)
Healthy fats 1 serving per day 2–3 serving per day >3 serving per day I don't use fats in my diet	980 (62.0%) 320 (20.3%) 85 (5.4%) 195 (12.3%)
Fast food 1–2 per month 1–2 per week 3–5 per week Daily I don't eat fast food	821 (52.0%) 517 (32.7%) 133 (8.4%) 23 (1.5%) 86 (5.4%)
Dairy products 1–2 per month 1–2 per week 3–5 per week Daily I don't eat dairy products	88 (5.6%) 312 (19.7%) 450 (28.5%) 696 (44.0%) 34 (2.2%)
High-fiber fruits 1–2 serving per day 3–4 serving per day 5 or more per day I don't eat high-fiber fruits	741 (46.9%) 84 (5.3%) 19 (1.2%) 736 (46.6%)
Leafy vegetables 1–2 serving per day 3–4 serving per day 5 or more per day I don't eat leafy vegetables	720 (45.5%) 91 (5.8%) 27 (1.7%) 742 (47.0%)
Drinking coffee 1–2 cups per month 1–2 cups per week 3–5 cups per week 1–2 cups Daily I don't drink coffee	94 (6.0%) 124 (7.8%) 162 (10.3%) 811 (51.3%) 389 (24.6%)
Drinking tea 1–2 cups per month 1–2 cups per week 3–5 cups per week 1–2 cups Daily I don't drink tea	182 (11.5%) 327 (20.7%) 239 (15.1%) 538 (34.1%) 294 (18.6%)
Supplements taken in the previous month Vitamin D Calcium Multivitamin Omega-3 (fish oil) Zinc Vitamin-C Iron Folic acid Other I don't take any supplement	575 (36.4%) 103 (6.5%) 337 (21.3%) 198 (12.5%) 187 (11.8%) 361 (22.8%) 75 (4.7%) 19 (1.2%) 18 (1.13%) 639 (40.4%)

According to dietary changes before or during PMS, most participants (65.7%) crave sweets. Moreover, the most common choices of herbal teas during PMS that the participants used were sage (34.4%), cinnamon (31.8%) and mint (27.0%), as shown in Table 5. Around 75% of participants reported using painkillers during PMS. Almost (45%) reported using pain killer once daily during their menstrual cycle. Concerning the type of painkiller, (46.8%) of the participants reported using paracetamol, more than two-fifths reported using a non-steroidal anti-inflammatory drug (NSAID) and (19.6%) did not use painkillers for pain management Table 5.

**Table 5.** Dietary behaviors before/during premenstrual syndrome and pain management of premenstrual syndrome.

Dietary behaviors before/during PMS	n (%)
Dietary changes during PMS No change Craving sweets (chocolate, cake, Eastern sweets) Craving savory snacks (nuts, potato chips, pickles) Craving pastries (pizza, croissants, pies) Other types	211 (13.4%) 1038 (65.7%) 522 (33.0%) 420 (26.6%) 322 (20.4%)
Use of herbal teas during PMS Cinnamon Mint Green tea Chamomile Rosemary Fennel Sage Anise Thyme I don't drink any type of herbals	503 (31.8%) 427 (27.0%) 242 (15.3%) 231 (14.6%) 152 (9.6%) 35 (2.2%) 544 (34.4%) 324 (20.5%) 52 (3.3%) 443 (28.0%)

Painkiller use during PMS	
Need of pain killer for PMS Yes No, I didn't need any	1189 (75.3%) 391 (24.7%)
Frequency of using pain killer Once daily 2 or more daily Often, I need to seek a hospital to control my pain I don't use pain killer	714 (45.2%) 430 (27.2%) 37 (2.3%) 399 (25.3%)
Type of painkiller NSAID's Paracetamol Other I don't use pain killer	681 (43.1%) 739 (46.8%) 231 (14.6%) 310 (19.6%)

NSAID: Non-steroidal anti-inflammatory drug; PMS: Premenstrual syndrome.

Further, there was an association between age and psychological and behavioral symptoms, where age was correlated with a decline in the likelihood of reporting psychological symptoms (OR: 0.17; 95% CI: 0.09–0.32; p < 0.05) and behavioral symptoms (OR: 0.34; 95% CI: 0.17–0.65; p < 0.05). Additionally, the likelihood of reporting psychological symptoms was increased with marital status (OR: 1.63; CI: 1.18–2.25; p < 0.05), in contrast, behavioral symptoms were decreased with marital status (OR: 0.62; CI: 0.5–0.8; p < 0.05). Moreover, PMS history was associated with psychological symptoms (OR: 2.4; 95% CI: 1.6–3.7; p < 0.05) and physical symptoms (OR: 2.9; 95% CI: 1.9–4.7; p < 0.05). Smoking status was related to a likely increase in reporting psychological symptoms (OR: 1.7; 95% CI: 1.3–2.6; p < 0.05), physical symptoms (OR: 3.1; 95% CI: 1.9–5.2; p < 0.05) and behavioral symptoms (OR: 2.2; 95% CI: 1.4–3.5; p < 0.05).

Surprisingly drinking tea was linked to a reduction in psychological, physical and behavioral symptoms. Moreover, it was found that females who crave special food were associated with raised psychological symptoms (OR: 3; 95% CI: 2.1–4.3; p < 0.05) and physical symptoms (OR: 5.1; 95% CI: 3.6–7.2; p < 0.05), and behavioral symptoms (OR: 1.7; 95% CI: 1.2–2.3; p < 0.05). Additionally, behavioral and physical symptoms rose with participants with higher BMI levels (1.5, 95% CI: 1.1–2.04; p < 0.05) and (OR: 2.01; 95% CI: 1.4–2.7; p < 0.05), respectively. Nonetheless, other variables, such as fast-food consumption, number of pregnancies and supplement use, were significantly associated with psychological symptoms. However, some variables were not statistically significant, such as coffee consumption and exercising shown in Table 6.

**Table 6.** Multiple regression analysis for dietary and lifestyle behaviors and associations with premenstrual syndrome.

Variable	OR	95% CI	p-value
Age	0.17	0.09–0.32	<.0001
BMI	1.1	0.78–1.5	0.6278
Married	1.63	1.18–2.25	0.0030
History of PMS	2.4	1.6–3.7	<.0001
Pregnancy history	0.59	0.43–0.83	0.0025
Smoking	1.7	1.3–2.6	0.0101
Exercise	1.2	0.87–1.64	0.2539
Complex carbohydrates	1.3	0.85–1.87	0.2378
Healthy fat	0.97	0.6–1.5	0.9116
Fast food	1.46	1.1–2.2	0.0195
Dairy product	0.52	0.23–1.01	0.0554
Fruits	0.76	0.56–1.0	0.0875
Green vegetables	0.87	0.64–1.2	0.3870
Coffee	1.1	0.79–1.5	0.5669
Herbal tea	0.45	0.32–0.62	<.0001
Supplement (yes)	1.5	1.1–2.1	0.0141
Vitamin-D	1	0.72–1.36	0.9128
Calcium	1	0.55–1.9	0.9656
Omega-3	1.1	0.66–1.82	0.7951
Multivitamin	1.6	1.0–2.5	0.0335
Vitamin C	0.75	0.51–1.1	0.1552
Zinc	1.4	0.83–2.6	0.1984
Food craving	3.0	2.1–4.3	<.0001
Use of analgesic	2.9	2.1–4.1	<.0001

Significant at p < 0.05.

CI: Confidence interval; OR: Odds ratio.

## Discussion

The initial purpose of the current study was to identify PMS prevalence and severity among Jordanian women, identify related sociodemographic factors and examine the correlation of PMS symptoms with dietary habits. Besides, it highlights the use of analgesics to relieve PMS pain. According to the current literature, this was the first study to examine the prevalence of PMS and its association with dietary habits among Jordanian women aged 18 to 50.

In the present study, age was significantly associated with a decreased risk of reporting psychological and behavioral symptoms of PMS. Mahin Delara *et al.* found similar results in an Iranian study, revealing a significant correlation between PMS and age [13]. In addition, our findings showed a positive association between PMS and family history, which aligns with earlier studies, illustrating that premenstrual symptoms were associated with a mother's PMS history [14–16]. This could be explained by shared biological and psychological factors influencing expectations and self-awareness [17,18].

The overall PMS prevalence was found to be (94%). The most frequently reported psychological and physical premenstrual symptoms were depressed mood (45.7%) and anger feelings (42.3%), muscle, joint, abdominal and back pain (43%), respectively, which is consistent with other studies, that observed how anger/irritability is the most frequently reported psychological symptom while abdominal pain was the most commonly observed physical symptom [19]. Moreover, in a Jordanian study, depression and mood swings were commonly reported PMS symptoms before menstruation [20]. However, another study found that the most common PMS symptoms were lethargy/fatigue, decreased energy, affective lability and depressed mood [21]. Additionally, in a study on Japanese women, Takeda *et al.* found that most women reported anxiety, anger, fatigue or lack of energy [22]. As noted, Chumpalova *et al.* evaluated the leading symptoms of PMS in Bulgarian women, including irritability, appetite changes, anxiety, fatigue, mood swings, abdominal bloating and tender breasts [23]. Despite the difference in the prevalence of PMS symptoms between the previous studies, the selection of sample size and the diagnostic tools will likely influence the results.

In this study, pregnancy history was significantly associated with PMS symptoms, decreasing the risk of reporting psychological symptoms. It has also been found that psychological and behavioral PMS symptoms are higher among single participants. In line with our findings, an earlier Jordanian study has demonstrated that PMS symptoms were more severe among married women [24]. Nonetheless, the current results are contrary to those of *Das's* group, which reported that unmarried women have a 5.9-fold higher risk of PMS than married women [25].

Based on anthropometric measurements, the average BMI of the participants was within the normal range. BMI was significantly associated with an increased risk of physical and behavioral symptoms of PMS. Our outcomes were consistent with previous findings, demonstrating significant correlations between obesity, BMI, and PMS [15,24,26]. These results have not confirmed previous research conducted by Aarushi Kharb *et al.*, who found no correlation between PMS and BMI [27]. Additionally, Isgin-Atici's study has reported insignificant differences in anthropometric measurements between PMS cases and their counterpart controls in Turkey [28].

Numerous studies have linked premenstrual symptoms with smoking [29,30]. In addition, a meta-analysis of 13 studies involving 25,828 participants found that smoking increases the risk of PMS (p = 0.0001) [31]. According to our results and previous research, adult female smokers have a significantly higher risk of reporting PMS than non-smokers. This could be explained by the effect of cigarette smoking on the dysregulation of estrogen, progesterone and gonadotropin levels, which may be involved in PMS development [30]. However, it is unclear whether smoking contributes to the etiopathogenesis of PMS or whether women suffering from PMS smoke as a means of relieving their symptoms [30].

Previously, it was found that carbohydrates and fiber consumption were not associated with PMS risk in a recent study by Houghton and colleagues, as well as a cross-sectional study conducted in the United Arab Emirates [4,32]. On the contrary, a study by Hussein and colleagues revealed that a high intake of carbohydrates was associated with premenstrual symptoms [33]. In our study, fast food consumption was positively related to psychological symptoms, similar to a study carried out in India has reported a significant association between frequent fast food consumption and PMS symptoms (p = 0.004) [34]. In contrast, Houghton found no correlation between fat intake and PMS [35].

This study's results reinforce Reem Abu Alwafa *et al.* study, which strongly recommended certain foods during menstruation [21]. Another study shows the desire for foods rich in sugar, salt and fat, such as chocolate, pastries, snacks, and desserts, was higher during the premenstrual period [36,37]. Moreover, according to Yukie Matsuura *et al.* under three-quarters (70.4%) of students had their appetites increased during menstruation cycles, but the highest number was observed before menstruation (85.8%) [38]. Further, Zellner *et al.* stated that American women were more likely to crave chocolate during the perimenstrual period than Spanish women [39]. This increase in carbohydrate consumption would also subjectively justify the increased need to consume food sources like chocolate, desserts, pastries and other foods. In theory, this may be explained by the relationship between simple carbohydrates (high glycemic index) and higher brain serotonin production [40], thus reducing negative mood effects [41].

In our study, we illustrated that PMS symptoms and analgesic use were significantly correlated, including the fact that three-quarters of participants needed analgesics to manage PMS-related pain. In agreement with our findings, participants in a cross-sectional study in Iran report that self-medication for PMS, especially using analgesics, is very common, at 70.2% [42]. Taken together, self-medication of PMS-related pain indicates the need for further research on properly using painkillers to relieve PMS symptoms.

We are aware that our paper has some limitations. First, participants were asked to recall some information that could affect the accuracy of the data; this is referred to as differential recall bias. Secondly, those inherent to any self-administered questionnaire study, such as the misclassification of answers whether related to intake of foods or symptoms. Furthermore, the sample primarily represented the middle and northern regions, with limited information available from the southern regions. Additionally, cross-sectional design limits the ability to infer causal relationships.

## Conclusion

As far as we know, the present paper is the first in Jordan to highlight the prevalence of PMS among females, examine PMS predictor factors in this population, and classify each premenstrual symptom according to severity. The results indicated a lack of significant association between PMS and dietary habits, while fast food consumption was found to be associated with psychological symptoms of PMS. To our expectations, food cravings were strongly related to the PMS domains. Additionally, herbal tea was found to have a protective effect against PMS. A significant correlation was found between PMS and several factors, including age, marital status, history of pregnancy and family history of PMS. Finally, most participants reported using painkillers during their PMS period.

**Summary points**

- Premenstrual syndrome (PMS) is highly prevalent among Jordanian women.
- Premenstrual syndrome affects women physically, psychologically and behaviorally.
- Dietary habits are strongly associated with the psychological symptoms of PMS.
- Family history, age, marital status, and history of pregnancy were associated with PMS.
- The use of painkillers was high during the PMS period as reported by respondents.
- Women need to identify their triggers/risk factors of PMS to minimize the burden associated with this condition.
